# Seroprevalence Patterns Suggestive of Postnatal *Trypanosoma cruzi* Acquisition in a Low-Infestation Area of Eastern Bolivia

**DOI:** 10.3390/tropicalmed11030070

**Published:** 2026-03-05

**Authors:** Beatriz Amparo Rodríguez-Olguin, Daniel F. Lozano Beltrán, Isabel Mariscal Sejas, Brandon N. Mercado-Saavedra

**Affiliations:** 1 Facultad de Ciencias Farmacéuticas y Bioquímicas, Universidad Autónoma Gabriel Rene Moreno, Santa Cruz 0002, Bolivia; 2 Fundación CEADES, Universidad Mayor de San Simón, Cochabamba 0001, Bolivia; 3 Hospital de Postrervalle, Postrervalle, Santa Cruz 0002, Bolivia; 4 Instituto de Investigación de Medicina, Universidad Católica Boliviana San Pablo, Santa Cruz 0002, Bolivia

**Keywords:** Chagas disease, vector-borne transmission, Bolivia, *Trypanosoma cruzi*

## Abstract

Chagas disease remains a major public health concern in Latin America, with Bolivia reporting one of the highest burdens of infection. While congenital transmission has become the predominant route of new infections in several countries, vector-borne transmission persists in rural and peri-urban regions. Postrervalle, in the department of Santa Cruz, is officially classified as a low-infestation area; however, updated community-level data remain limited. We conducted a cross-sectional study in July 2023 involving 58 mothers and 104 of their children in Postrervalle. Participants underwent serological screening with three diagnostic assays, and epidemiological data were collected via structured maternal questionnaires. Logistic regression models were used to assess associations between child *Trypanosoma cruzi* seropositivity and maternal or household exposures during pregnancy. Seroprevalence was 15.5% among mothers and 3.8% among children. Notably, all seropositive children were born to mothers who tested seronegative, suggesting non-congenital transmission. In multivariable analysis, living in houses with mud walls during pregnancy was strongly associated with child seropositivity (adjusted OR = 38.566), while older child age also increased the odds of infection. Other maternal exposure variables showed elevated but imprecise associations. Despite its classification as a low-infestation area, Postrervalle shows serological patterns consistent with postnatal acquisition linked to domestic structural conditions that facilitate triatomine colonization. These findings overall highlight the need for integrated serological and entomological surveillance to better characterize and prevent Chagas transmission in rural communities.

## 1. Introduction

Chagas disease (CD) is caused by the protozoan *Trypanosoma cruzi* (*T. cruzi*) and is prevalent in most Latin American countries [1,2,3]. It is estimated that over 7.5 million people are currently infected with the parasite, and more than 70 million people are at risk of infection [1,4]. CD has different modes of transmission, including vector-borne, congenital, and by blood transfusion [1,3]. It is estimated that 1 out of 3 people infected with the parasite might progress to Chagas Cardiomyopathy, one of the chronic manifestations of CD [5,6,7]. Across Latin America, all transmission routes have been documented, but the dominant pathway has increasingly shifted from vector-borne transmission to congenital transmission in many settings [3,4,5]. Among these countries, Bolivia is particularly affected, presenting both vector and congenital transmission, making it a critical context for the study of CD [2,3,5].

Despite advances in control across the region, CD remains a neglected infectious disease, and Bolivia continues to carry one of the highest burdens worldwide [8]. The high prevalence of CD in Bolivia might be due to the vector’s presence across several areas of its territory, affecting over 40% of the country’s communities, according to the Bolivian National Program of Chagas disease (BNPCD) [9]. A research study evaluated the prevalence of CD in a rural community in the Gran Chaco, a region highly endemic for the vector in Bolivia, reporting a prevalence of 40–80% [10]. This high endemicity is also reflected among Bolivian migrants, who contribute disproportionately to CD cases identified in non-endemic countries. A community-based study conducted in Spain among people from Latin American countries found that 40% of participants were from Bolivia, and 36% were seropositive for CD [11]. Altogether, these data highlight the substantial burden of CD in Bolivia; moreover, as the relative importance of transmission has shifted in many Latin American and non-endemic settings from vector-borne to congenital transmission, understanding CD prevalence among women of reproductive age is crucial to guide screening and prevention strategies.

Recent evidence suggests that, as of 2020, approximately 12% of pregnant women in South America are infected with *T. cruzi* [12]. In Bolivia, data from the BNPCD reported a prevalence of 2.4% in children aged 5 to 15 years and a congenital transmission rate of 1.4% in 2019 [13]. By 2022, the National Program of Vector-Borne Diseases (ETVs) estimated a chronic infection prevalence of 19.2% among individuals aged 15 years or older [14]. Although vector control initiatives and expansions in maternal and child health care have led to declines in severe morbidity and mortality associated with CD in Bolivia, evidence of a sustained reduction in vector-borne transmission remains inconclusive [12,13]. Subsequent surveillance data have not consistently confirmed the decreases reported in earlier program evaluations [15]. A 2017 study conducted in Camiri, a municipality in the Gran Chaco region of Bolivia, confirmed a seroprevalence of 0.22% among children aged 5 to 21 years, with rates increasing with age and a mean age of seropositivity of 13 years [16]. Similarly, studies in urban areas of Cochabamba reported prevalence rates ranging from 19% to 25% among children aged 5 to 13 years, reinforcing the notion that CD remains not only a rural but also an urban public health concern [17]. Together, these heterogeneous findings highlight persistent uncertainty about the relative contribution of vector-borne versus congenital transmission across Bolivian settings, particularly in areas classified as low infestation.

This study aims to describe the epidemiological characteristics of CD among mothers and their children in the community of Postrervalle, a locality officially classified by the Bolivian National Chagas Program as having a low vector infestation rate [9]. By examining seroprevalence patterns and associated risk factors, this study aims to assess whether infections in children are more consistent with postnatal acquisition than congenital transmission, and to identify household factors associated with child seropositivity.

## 2. Methods

### 2.1. Study Population

**Figure 1:**
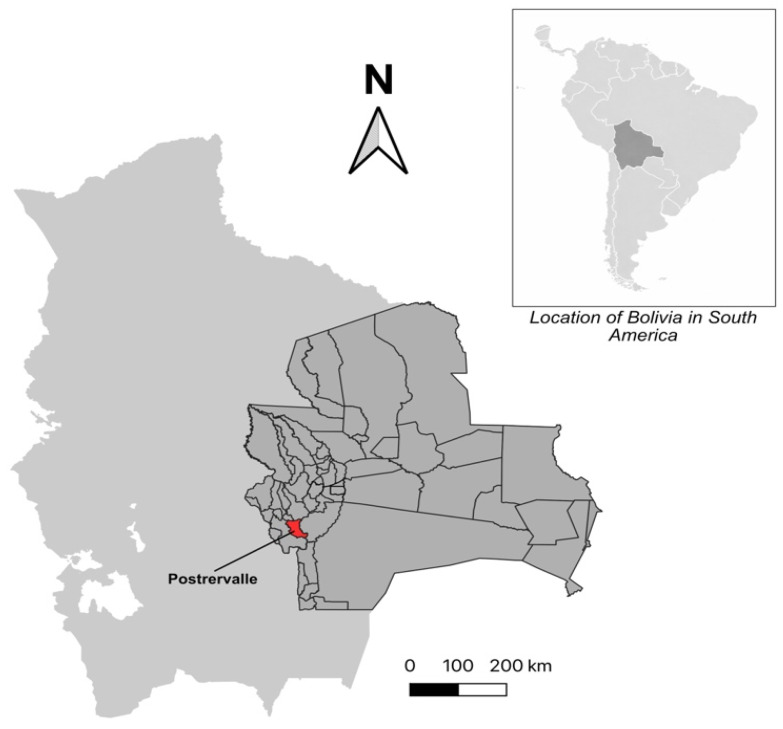
Location of Postrervalle in Santa Cruz, Bolivia. The upper right corner map shows the location of Bolivia in South America. The map of Bolivia in light gray shows the location of the department of Santa Cruz (dark gray) and its communities. Postrervalle is located in the southwest of Santa Cruz.

This is a cross-sectional, non-probabilistic, exploratory study of mother–child pairs. The study population included 58 mothers and their children (104 in total), aged 4 to 17 years. The recruitment took place in July 2023 in the Postrervalle municipality, located in the southwest of the Santa Cruz department, Bolivia (Figure 1). According to the 2024 national census [18], Postrervalle has a population of 1804 inhabitants. Of these, 528 are children and adolescents aged 0–17. The municipality has only two schools, one elementary school and one high school, with a combined enrollment of 342 students. The study population consisted of students from these two schools. All students with their mothers were invited to participate in the study. A total of 58 mothers and 104 students (children) agreed to participate in the study; 17 mothers recruited had one child, 27 had two children, 10 had 3 children, and 4 had 4 children. Nine children refused to participate, despite their mother and siblings participating in the study.

The recruitment began with a presentation of the study to all parents at a meeting convened by the school head. Following this meeting, both school authorities and the parents’ representatives agreed to allow the study to be conducted on the school premises during the following week. Mothers were then invited to attend the school on a scheduled date with their children to participate in the study.

All participating mothers completed an epidemiological questionnaire that included sociodemographic information, details about household characteristics (such as wall materials), and questions related to each of their pregnancies (including Chagas testing, history of blood transfusions, and presence of kissing bugs in the home). The pregnancy-related section was administered separately for each pregnancy reported by the mother during the questionnaire.

### 2.2. Specimen Collection and Sample Processing

Venous blood samples of 3 mL and 5 mL in volume were collected from consenting students and their mothers, respectively. Samples were allowed to clot and were centrifuged at 3500 rpm within the first hour of collection to separate serum. Serum was aliquoted into 1.5 mL cryovials to minimize freeze–thaw cycles and stored at −20 °C until analysis; each aliquot underwent a single freeze–thaw cycle before testing.

Aliquots were tested using three serological assays to detect anti-*Trypanosoma cruzi* IgG antibodies: OnSite Chagas Ab Combo Rapid Test—CTK BIOTECH (Poway, CA, USA), Chagatest HAI—Wiener lab (Rosario, Argentina), and Chagatest ELISA recombinant v.3.0—Wiener lab (Rosario, Argentina). The OnSite rapid test was interpreted visually as reactive/non-reactive based on the presence of the test line. For Chagatest HAI and Chagatest ELISA recombinant v3.0, results were interpreted using the manufacturer-recommended cutoffs. Each run included manufacturer-provided positive and negative controls (and calibrators when applicable), and results were accepted only when control values met the specified validity criteria.

In accordance with the WHO recommendations for chronic Chagas infection diagnosis, which require concordant positivity in at least two serological assays, participants with ≥2 positive tests were classified as *T. cruzi* seropositive; those with only one positive result were classified as seronegative [19]. All seropositive participants (children and their mothers) were referred to the National Program for Chagas in the municipality for follow-up and treatment in accordance with national regulations.

### 2.3. Statistical Analysis

The data were analyzed using Stata 19.0 (StataCorp, College Station, TX, USA). Continuous variables are summarized as means ± standard deviations (SDs) or medians with interquartile ranges (IQRs), depending on their distribution, assessed by the Shapiro–Wilk test. Categorical variables are expressed as absolute frequencies and percentages. Comparisons between Chagas-seropositive and -seronegative groups were conducted separately for mothers and children. For continuous variables, Student’s *t*-test was applied when normality and homoscedasticity assumptions were met, while the Mann–Whitney test was used for non-normally distributed data. For categorical variables, the chi-square test or Fisher’s exact test (when expected cell counts were <5) was used to assess associations between serological status and epidemiological factors.

The primary outcome was *T. cruzi* seropositivity, defined as reactivity in at least two of the three serological assays performed. Independent variables included demographic factors (e.g., maternal age, number of children), housing characteristics (wall, roof, and floor materials, fumigation history), and exposure-related variables (history of blood transfusion, presence of triatomine bugs, being bitten by kissing bugs, and previous Chagas testing or treatment). Given the small number of seropositive children, we used Firth’s penalized logistic regression as the primary analysis. Because children were clustered within mothers, we also performed a sensitivity analysis using logistic regression with standard errors clustered by mother.

All statistical tests were two-tailed, and a *p*-value < 0.05 was considered statistically significant. Missing data were minimal (<5%) and excluded from specific analyses as pairwise deletions. Graphical representations (age distribution of seropositive vs. seronegative children) were produced using a violin plot to visualize distributional differences.

### 2.4. Ethical Aspects

The research protocol was reviewed and approved by the Institutional Review Board (IRB) of the Bolivian Catholic University in Santa Cruz (approval code 032; approved on 26 July 2022). Recruitment and data/sample collection were conducted in Postrervalle between 24 July 2023 and 27 July 2023, following prior coordination meetings with local authorities and school representatives. Written informed consent was obtained from all participating mothers for their own participation and for their child’s participation; children also provided assent. Participants with serological evidence of *T. cruzi* infection were referred to the municipal National Chagas Program for clinical evaluation, follow-up, and management according to national regulations.

## 3. Results

### 3.1. Demographic Characteristics of Mothers

The study included 58 mothers, of whom 49 (84.5%) were seronegative for *T. cruzi*, and 9 (15.5%) were seropositive for *T. cruzi* (Table 1). The median age of the mothers was 37 years (IQR: 32–42), with no significant difference between the seronegative and seropositive groups (*p* = 0.494). Both seropositive and seronegative mothers had a median number of 2 children (*p* = 0.463). Although 49 (86%) of mothers reported having been tested for CD at some point in their lives, the proportion was slightly higher among seropositive mothers (88.5%)—even though the difference was not significant (*p* = 0.783). Nevertheless, the same number of mothers (4) from each group (seropositive and seronegative) reported being bitten by a kissing bug; the proportion was significantly higher in seropositive mothers 4 (44.4%) compared to seronegative mothers 4 (8.3%; *p* = 0.004). Additionally, seropositive mothers were more likely to receive Chagas treatment (22.2% vs. 2.1%, *p* = 0.014).

### 3.2. Chagas Serology in Mothers and Children

Chagas serology testing was performed on 58 mother–child groups. Each mother was paired with all their children included in the study. As mentioned in Table 1, the prevalence of Chagas seropositivity among mothers was 15.5%. Table 2 describes that 6.9% of mothers had seropositive children. All seropositive children were born to seronegative mothers; in all cases, all three serological tests were negative for mothers and positive for their children. Among children of seronegative mothers, 8.2% were seropositive, though this difference was not statistically significant.

### 3.3. Factors Associated with Child Trypanosoma Cruzi Seropositivity

**Figure 2:**
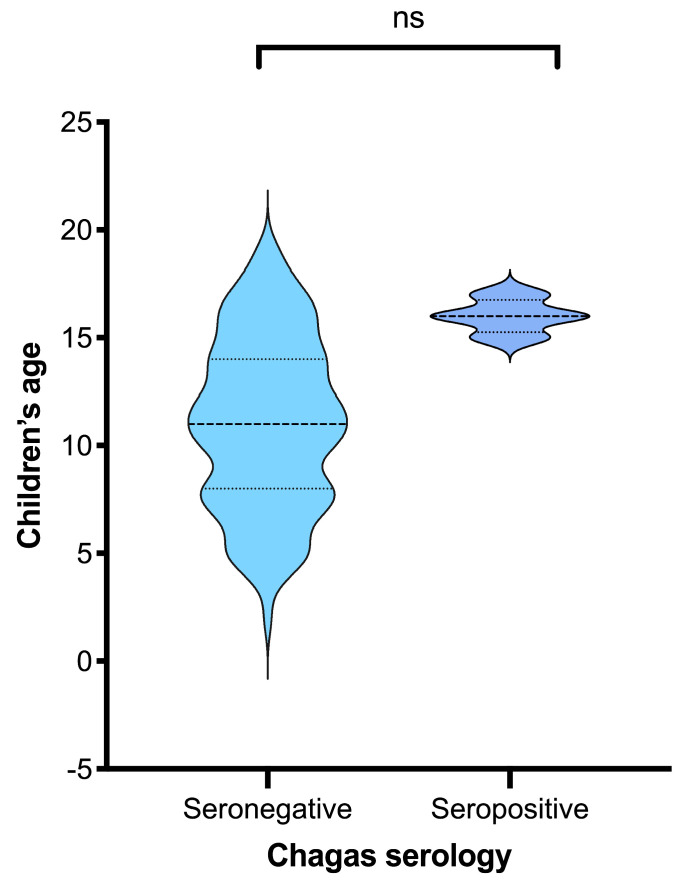
Age distribution of seropositive and seronegative children (N = 104). Children’s age distribution by Chagas serostatus. The non-parametric Mann–Whitney’s *p* value is 0.083 (ns), suggesting a non-significant difference.

A total of 104 children were included in the analysis, of whom 96.2% were seronegative, and 3.8% were seropositive for Chagas. Seropositive children were clustered at higher ages than seronegative children, whose ages were broadly distributed, although this difference was not significant (*p* = 0.083) (Figure 2). Firth’s logistic regression analyses were conducted to identify factors associated with *T. cruzi* seropositivity among children, using epidemiological information reported by their mothers regarding exposures during pregnancy (Table 3). In the unadjusted models, child age was significantly associated with seropositivity (OR = 1.587; 95% CI: 1.052–2.395; *p* = 0.028), indicating that the likelihood of infection increased with age. No statistically significant associations were found with sex or being born in an urban setting.

Among exposure variables reported during pregnancy, two maternal factors showed elevated odds in unadjusted analyses: previous Chagas treatment (OR = 28.428; 95% CI: 2.329–346.893; *p* = 0.009) and living in a house with mud walls (OR = 8.628; 95% CI: 1.202–61.941; *p* = 0.032). Although wide confidence intervals reflect small subgroup sizes, these findings suggest that domestic structural conditions and prior maternal infection history may be linked to child seropositivity.

After adjustment for child age and sex, the association between living in a house with mud walls and child seropositivity remained statistically significant (adjusted OR = 38.566; 95% CI: 1.897–783.894; *p* = 0.017). The relationships with maternal Chagas treatment, blood transfusion, and reported contact with kissing bugs showed elevated but non-significant adjusted odds ratios. In sensitivity analyses accounting for within-mother clustering (57 mother clusters), associations were generally similar in direction. However, confidence intervals remained wide because of the small number of seropositive children (Supplementary Table S1). This is because the analysis used standard logistic regression with clustered SEs rather than penalized regression and therefore yields different point estimates under sparse data.

## 4. Discussion

Latin American countries have several neglected diseases, such as CD, with approximately 70 million people at risk of infection. Congenital transmission is the main source of infection in most countries in the region [20,21]. Bolivia is a country with one of the highest burdens of CD, where multiple rural communities in the Chaco region are endemic to the vector [8,9,22]. The official data presented by the BNPCD reports that Postrervalle is a low-infestation community due to the low infestation rate of vectors (<1%) [14]. The BNPCH also reports that *Triatoma infestans* is the principal vector present in the region. In this study, we describe the prevalence of CD among mothers and children in Postervalle, with a prevalence of 15.5% among mothers. This prevalence is similar to that reported by local authorities, which estimates it at 14.5% (unpublished official data). However, a recent meta-analysis described that the prevalence of pregnant women in Bolivia ranged from 17% to 65%, with lower rates in the past 5 years [23]. This relates to a study that reports that the prevalence among pregnant women at the public maternity hospital in Santa Cruz is 22% [24]. Nevertheless, these results were mainly hospital-based, with women from all regions of the department (rural and urban), thereby increasing the estimated prevalence within each community. One factor that was significantly more prevalent among Chagas seropositive mothers in this study was being bitten by the vector, with 4 (44%) of infected women reporting being bitten by a kissing bug. However, having lived in rural areas and living in houses with mud walls was not significantly more prevalent in women with CD. These results are not entirely consistent with other studies conducted in similar settings [10,11,25], which may be due to the small sample size or to the fact that most of the people included in the study have lived in Postrervalle for their entire lives, presenting similar epidemiological characteristics. One interesting finding was the case of a seronegative mother who had previously received treatment for CD. In this study, the mother tested negative across all three serological assays, which makes a current false-negative result unlikely. While treatment-induced seroconversion can occur, it is rare in chronically infected adults and typically requires many years of follow-up before antibody levels fall below detectable thresholds; it is most observed in infants or young children treated early in the course of infection [26]. Therefore, given the mother’s adult age and the complete seronegative profile at the time of evaluation, the most plausible explanation is that she likely received a false-positive diagnosis in the past, rather than having undergone treatment-induced loss of detectable antibodies. These findings highlight the importance of contextualizing local seroprevalence patterns within broader regional transmission dynamics and historical vector control efforts.

Bolivia joined the Southern Cone Initiative, a 1991 collaborative program with Argentina, Brazil, Chile, Paraguay, and Uruguay, which aimed to interrupt transmission of *T. cruzi* by eliminating *Triatoma infestans* and ensuring safe blood supplies [27]. While countries like Uruguay, Chile, and Brazil successfully halted *Triatoma infestans* transmission by the late 1990s and early 2000s, Bolivia achieved interruption in parts of La Paz and Potosí only by around 2011–2013. However, persistent bug populations and insecticide resistance continue to challenge complete eradication in regions such as the Gran Chaco [28]. Notably, in our study, none of the mothers who were seropositive for *T. cruzi* had seropositive children, whereas all seropositive children were born to mothers who tested negative. In all cases, the three serological tests were consistently negative for the mothers and positive for the children, making false-negative or false-positive results unlikely. Because congenital transmission occurs only from infected mothers, this pattern indicates that infections in the children in our study are unlikely to be vertically acquired. Instead, these findings are more consistent with ongoing vector-borne exposure in the domestic environment, a pattern also documented in childhood serosurveys in the Bolivian Chaco [16,29].

In Postrervalle, congenital transmission may be minimal, and vector-borne transmission is likely the source of infection among children. Although maternal screening was conducted at the time of this study, some mothers may have seroconverted after giving birth, or some results may have been false negatives due to the limited sensitivity of serological tests during the acute or early chronic stages of infection. Alternatively, postnatal vector exposure is a plausible explanation, as all seropositive children were older than 8 years. This epidemiological pattern aligns with other studies conducted in rural or peri-urban areas of Bolivia with similar vector infestation classifications. For instance, Hopkins et al. (2019) reported persistent *T. cruzi* transmission in school-aged children in the Bolivian Chaco, even in areas under surveillance, with infection rates increasing with age and suggesting ongoing vector exposure in domestic settings [16]. In another low-endemic setting in Cochabamba, Medrano-Mercado et al. (2008) found seroprevalence rates in children as high as 25%, reinforcing that low infestation classifications may not fully reflect the true risk of transmission, particularly in settings with structural vulnerabilities such as adobe walls and a lack of sustained vector control [17]. Our logistic regression findings (Table 3) further support the role of domestic environmental conditions in transmission. Age was significantly associated with *T. cruzi* seropositivity among children. All seropositive cases occurred in children aged 15 years or older, while seronegative children showed a broader age distribution, suggesting cumulative exposure with increasing age. Most notably, living in a house with mud walls during pregnancy remained significantly associated with seropositivity after adjustment, indicating that household construction type may serve as a persistent ecological risk factor enabling vector colonization. Results were directionally consistent in a sensitivity analysis that accounted for within-mother clustering using cluster-robust standard errors (Supplementary Table S1). However, confidence intervals remained wide due to the small number of seropositive children. Although other exposure-related variables (such as a maternal history of kissing bug contact or prior Chagas treatment) showed elevated odds ratios, their wide confidence intervals likely reflect the small number of seropositive children and should be interpreted cautiously. Additionally, the potential emergence of pyrethroid resistance in *Triatoma infestans*, the principal domestic vector in Bolivia, is a growing concern. Resistance has been documented across several regions of the Gran Chaco, reducing the long-term effectiveness of indoor residual spraying campaigns [30]. If similar resistance is present in Postrervalle, even a low measured infestation prevalence may mask ongoing domestic colonization and transmission. This reinforces the need to integrate routine entomological surveillance, community-based vector monitoring, and repeated serological screening of children—not only infestation-level classifications—to assess transmission risk accurately. Finally, the characteristics associated with seropositivity in children included whether their mothers had seen or been bitten by kissing bugs and whether they had lived in houses with mud walls, environments that likely reflect the domestic setting in which the children were born and spent their early childhood. These conditions represent key risk factors that facilitate vector-borne transmission of *T. cruzi* [24,31]. The observed serological pattern is consistent with postnatal acquisition and warrants strengthened surveillance; however, we did not collect contemporaneous entomological data to demonstrate ongoing domestic vector transmission directly. Taken together, these findings highlight that domestic ecological conditions continue to play a central role in sustaining vector-borne transmission, even in communities labeled as low-risk.

This study has limitations, including a relatively small sample size, non-probabilistic sampling, and incomplete inclusion of all children from participating mothers. Recruitment was conducted through the two schools in Postrervalle, and participation depended on school attendance and caregiver availability on recruitment days; therefore, selection bias is possible. Children who were not enrolled, absent, or whose guardians could not attend may differ systematically in housing conditions and mobility/migration history, which could affect prevalence estimates and associations. The high school coverage in the community may partly mitigate the potential magnitude of this bias. According to the most recent census, 94.4% of children are registered in one of the two Postervalle schools, suggesting that the sampling frame encompassed most school-aged children. Nevertheless, only ~30% of registered students participated, and residual selection bias cannot be ruled out; thus, the findings should be interpreted as community-based rather than fully representative. In addition, not all children of enrolled mothers were included (primarily because verbal consent could not be obtained on the recruitment dates). Because siblings may share household environments and exposure risks, this within-family nonparticipation may have a limited impact on the direction of associations, but it could still influence point estimates of prevalence and effect sizes. Finally, the very small number of seropositive children led to wide confidence intervals, and the regression estimates should be considered exploratory and hypothesis-generating rather than causal.

These findings highlight that even in areas officially classified as low infestation zones, vector-borne transmission of *T. cruzi* remains a route of infection among children. While Bolivia has made substantial progress under the Southern Cone Initiative, particularly in selected regions, the continued presence of infected vectors and active transmission in communities like Postrervalle underscores the fragility of these gains. Moreover, the overuse of insecticides—potentially leading to vector resistance—and the broader ecological impacts of climate change may further hinder vector control efforts, threatening to reverse recent advances and sustain transmission in vulnerable rural populations.

## Figures and Tables

**Table 1:** Demographic and exposure characteristics of mothers stratified by Chagas serology status (N = 58).

Variables	Serology Status for Chagas in Mothers			
	Negative 49 (84.5%)	Positive 9 (15.5%)	Total 58 (100%)	*p* Value
Mother’s age	37 (32–41)	38 (36–44)	37 (32–42)	0.494 ^!^
Number of children	2 (1–3)	2 (2–2)	2 (1–2)	0.463 ^!^
Chagas test (yes) *	41 (85.4%)	8 (88.9%)	49 (86.0%)	0.783
Received blood transfusion (yes) *	5 (10.4%)	0 (0.0%)	5 (8.8%)	0.311
Seen kissing bugs (yes) *	7 (14.6%)	3 (33.3%)	10 (17.5%)	0.175
Lived in rural areas (yes) *	18 (37.5%)	1 (11.1%)	19 (33.3%)	0.123
Been bitten by a kissing bug (yes) *	4 (8.3%)	4 (44.4%)	8 (14.0%)	0.004
Received treatment for Chagas (yes) *	1 (2.1%)	2 (22.2%)	3 (5.4%)	0.014
House received fumigation *	31 (64.6%)	8 (88.9%)	39 (68.4%)	0.150
Mud wall (yes) *	13 (27.1%)	2 (22.2%)	15 (26.3%)	0.761

* During any pregnancy. ^!^ Mann–Whitney test. All other tests were performed using the X^2^ Fisher’s exact test. Numeric variables (mother’s age and number of children) are represented by Median (IQR).

**Table 2:** Chagas serology results of mothers and their children (N = 58).

	Mothers’ Chagas Serology		
	Negative (%)	Positive (%)	Total
Children’s Chagas serological status			
Negative	45 (91.8%)	9 (100.0%)	54 (93.1%)
Positive	4 (8.2%)	0 (0.0%)	4 (6.9%)
Total	49 (84.5%)	9 (15.5%)	58 (100%)

**Table 3:** Associated factors with *Trypanosoma cruzi* seropositivity among children, based on maternal and household exposures during pregnancy (N = 104).

Variables		Unadjusted			Adjusted		
		OR	[95% CI]	*p* Value	OR	[95% CI]	*p* Value
Age		1.587	[1.052–2.395]	0.028	1.615	[1.076–2.429]	0.021
Child sex (female)		1.433	[0.237–8.650]	0.694	2.779	[0.376–20.525]	0.316
Lived in rural setting		5.854	[0.823–41.615]	0.077	18.163	[0.947–348.212]	0.054
Having a seropositive mother		0.712	[0.0362–13.981]	0.823	0.079	[0.001–6.239]	0.255
During pregnancy, mother							
	received blood transfusion	7.285	[0.895–59.327]	0.063	37.858	[0.736–1948.268]	0.071
	saw kissing bugs in the house	3.622	[0.441–29.715]	0.231	5.316	[0.483–58.469]	0.172
	was bitten by a kissing bug	5.274	[0.625–44.492]	0.126	7.711	[0.614–96.780]	0.114
	received Chagas treatment *	28.428	[2.329–346.893]	0.009	10.864	[0.639–184.793]	0.099
	live in house with mud wall (yes)	8.628	[1.202–61.941]	0.032	38.566	[1.897–783.894]	0.017

Firth’s penalized likelihood logistic regression; * refers to treatment before pregnancy. Adjusted by age and sex.

## Data Availability

The curated dataset supporting the findings of this study is available in the Supplementary Materials of this article.

## Supplementary Materials

The following supporting information can be downloaded at: https://www.mdpi.com/article/10.3390/tropicalmed11030070/s1, Table S1: Sensitivity analysis of factors associated with child *Trypanosoma cruzi* seropositivity using logistic regression with robust standard errors clustered by mother.
