# Supplementary material for: Seroprevalence Patterns Suggestive of Postnatal Trypanosoma cruzi Acquisition in a Low-Infestation Area of Eastern Bolivia
Source: Trop Med Infect Dis. 2026 Mar 5;11(3):70. doi: 10.3390/tropicalmed11030070 (PMC13029981; doi:10.3390/tropicalmed11030070)
Supplement: Supplementary file 1 [file tropicalmed-11-00070-s001.zip › tropicalmed-4140099-supplementary/Supplementary Table S1.docx]

Supplementary Table S1. Sensitivity analysis of factors associated with child *Trypanosoma cruzi* seropositivity using logistic regression with robust standard errors clustered by mother (N = 104).

| **Variables** | | **Unadjusted** | |  | **Adjusted** | |  |
| --- | --- | --- | --- | --- | --- | --- | --- |
|  |  | **OR** | **[95% CI]** | ***p* Value** | **OR** | **[95% CI]** | ***p* Value** |
| Age |  | 1.724 | [1.326–2.241] | <0.001 | 1.799 | [1.276–2.535] | 0.001 |
| Sex (women) | | 1.439 | [0.184–11.241] | 0.729 | 3.182 | [0.222–4.591] | 0.394 |
| Lived in rural setting | | 7.607 | [0.773–74.869] | 0.082 | 87.268 | [1.579–4821.17] | 0.029 |
| During pregnancy, mother | | |  |  |  |  |  |
|  | received blood transfusion | 6.20 | [0.764–50.306] | 0.088 | 89.999 | [2.980–2718.237] | 0.010 |
|  | saw kissing bugs in the house | 3.115 | [0.290–33.423] | 0.348 | 7.469 | [0.350–159.429] | 0.198 |
|  | was bitten by a kissing bug | 4.611 | [0.443–47.966] | 0.201 | 11.474 | [0.760–173.335] | 0.078 |
|  | received Chagas treatment | 33.0 | [1.601–680.271] | 0.024 | 16.327 | [0.335–794.979] | 0.159 |
|  | live in house with mud wall (yes) | 11.285 | [1.195–106.547] | 0.034 | 225.44 | [1.807–28131.6] | 0.028 |
| Logistic regression with robust standard errors clustered at the mother level (n = 104 children with non-missing serostatus; 58 mother clusters).  Maternal serostatus (‘having a seropositive mother’) was not included in the clustered logistic regression sensitivity analysis because of complete separation (perfect prediction), resulting in non-estimable odds ratios in standard logistic regression. | | | | | | | |
| Adjusted by age and sex | |  |  |  |  |  |  |
